# Photocatalytic production of dihydroxyacetone from glycerol on TiO_2_ in acetonitrile†

**DOI:** 10.1039/c9ra09434b

**Published:** 2020-01-30

**Authors:** Alexander Luis Imbault, Jianyu Gong, Ramin Farnood

**Affiliations:** Department of Chemical Engineering and Applied Chemistry, University of Toronto Toronto Ontario M5S 3E5 Canada ramin.farnood@utoronto.ca; School of Environmental Science and Engineering, Huazhong University of Science and Technology Wuhan China

## Abstract

In this paper, photocatalytic production of dihydroxyacetone (DHA) from glycerol in acetonitrile on TiO_2_ was investigated. HPLC-MS analysis showed that glycerol was converted to DHA, glyceraldehyde (GAD), glyceric acid and several other chemicals. Using acetonitrile as the reaction medium instead of water not only provided a more selective process for production of DHA but also increased the glycerol conversion. After 300 min, with 1 g L^−1^ catalyst loading and 4 mM initial glycerol concentration, glycerol conversion and DHA selectivity were 96.8% and 17.8% in acetonitrile compared to 36.1% and 14.7% in water, respectively. The half-life of glycerol decreased by a factor of 6.2, from 467 min to 75 min, by changing the solvent from water to acetonitrile. Experiments using biodiesel-derived crude glycerol verified the effectiveness of the proposed process for the photocatalytic production of DHA from crude glycerol. A mechanism was proposed to explain the higher selectivity towards DHA over GAD in this process.

## Abstract 2

Photocatalytic conversion of glycerol and selectivity for dihydroxyacetone production was increased by using acetonitrile as the reaction medium.

## Graphical abstract

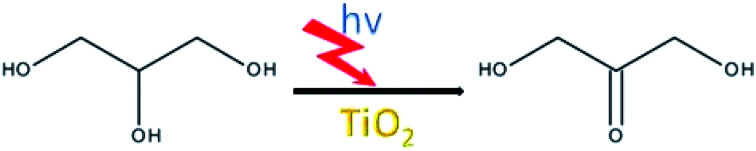

## 1. Introduction

Solar-to-chemical (STC) technologies in which solar radiation is utilized to generate valuable chemicals from organic waste streams, is an effective strategy to lower the environmental footprint of industrial processes.^1,2^ One such waste stream that is produced in large quantities is crude glycerol. Crude glycerol is generated during biodiesel production and is generally disposed of by burning.^3^ In addition, glycerol can be used as the building block for various fine chemicals such as dihydroxyacetone (DHA).

DHA is considered a platform chemical and is used in cosmetics, sunless tanning formulas, as well as food and pharmaceutical industries.^4–6^ Commercially, DHA is produced by fermentation of purified glycerol.^7,8^ However, given the higher value of DHA compared to glycerol (150 US$ per kg *vs.* 0.30 US$ per kg) attempts were made to develop alternative processes for DHA production using TEMPO, hydrogen peroxide, thermo-, electro- and heterogeneous catalysis.^5,8,9^ Notably, Hirasawa *et al.* utilized a Pd–Ag catalyst to synthesize DHA from glycerol in liquid water with 82% selectivity at elevated pressure and temperature under oxygen atmosphere (0.3 MPa O_2_ and 353 K).^10^ The high selectivity of this process towards DHA was attributed to the steric favourability of the adsorption of a primary hydroxyl group over the secondary hydroxyl group by the catalyst. In the mechanism proposed by Hirasawa *et al.*, the hydroxyl group adsorbed on the catalyst surface was protected from oxidation, instead the vicinal hydroxyl group was oxidized. According to this mechanism, DHA was formed by adsorbing the sterically favoured primary hydroxyl, whereas GAD would be formed by adsorbing the sterically inhibited secondary hydroxyl.^11,12^

DHA has been also produced from glycerol using heterogeneous photocatalysis in aqueous medium. Augugliaro *et al.* examined a range of UV irradiation, TiO_2_ catalyst loadings and glycerol concentrations to generate DHA in water and reported a maximum DHA selectivity of 8% at a conversion of 35% after 70 h of irradiation.^13^ Similarly, using Pd/TiO_2_ photocatalyst for the oxidation of glycerol in water, Zhou *et al.* reported selectivities as high as 9% for DHA production and a glycerol conversion of 21% after 18 h reaction time.^14^

A possible approach to increase the DHA yield in photocatalytic oxidation of glycerol is by selecting a suitable solvent. The choice of solvent affects valence and conduction bands and hence the reactivity and selectivity of photocatalysts.^15,16^ Additionally, changing the solvent alters the affinity of reactants and intermediate species to adsorb to the photocatalyst surface. Fox *et al.* investigated using different solvents to manipulate the oxidation of 1,4-pentanediol. By finding a solvent that facilitated the adsorption of a primary alcohol but not a secondary alcohol to the photocatalyst, they were able to maximize the formation of 4-hydroxypentanal and prevented its further oxidation.^17^ If the photocatalyst adsorbed both primary and secondary alcohols, a carboxylic acid would have been produced, not an aldehyde. Similarly, benzyl alcohol and its derivatives can be oxidized to their corresponding benzyl aldehydes *via* visible light photocatalysis in water as well as several non-aqueous solvents including acetonitrile and toluene.^18–20^ It has been reported that the selectivity of this reaction is higher in acetonitrile than in water.^21,22^

This paper offers a novel and more selective method for the photocatalytic production of DHA from glycerol by using acetonitrile as the reaction medium. Discovering value added uses for crude glycerol, a waste product from biodiesel production, offers a large boon. This is further compounded because glycerol produced from biodiesel is commonly disposed of by burning that is environmentally unfriendly.

## 2. Experimental

### 2.1. Materials and chemicals

Degussa P25 titanium dioxide, titanium(iv) isopropoxide (97%) and glycerol (>99%) were purchased from Sigma-Aldrich (Mississauga, Canada). Crude glycerol sample from biodiesel production was obtained from Biox Corporation (Hamilton, Canada). Hydrochloric acid (reagent grade, 12 M) was purchased from BioShop (Burlington, Canada). Ethanol (99%, anhydrous) was purchased from Commercial Alcohols (Toronto, Canada). Methyl orange (ACS reagent grade) was purchased from Fluka (Oakville, Canada). 2,4-Dinitrophenylhydrazine (DNPH) mixed in 30% water by weight was purchased from Spectrum Chemical MFG Corp (New Brunswick, United States of America). All chemical reagents were of analytical grade and used without further purification (except DNPH which was purified as described in Section 2.3). Ultrapure water (resistivity > 18 MΩ cm) obtained from a water purification system (Millipore, Etobicoke, Canada) was used. 0.2 μm PTFE filters and silica C18 3 mL 500 mg bed SPE columns were purchased from VWR.

### 2.2. Photocatalyst preparation

The synthesis of TiO_2_ was performed through a hydrothermal autoclave method. 15 mL of anhydrous ethanol was mixed with 5 mL of titanium(iv) isopropoxide in a 50 mL Teflon container. 1 mL of 6 M hydrochloric acid in 5 mL of anhydrous ethanol was added to the previous mixture dropwise over the course of 5 min. The mixture was then stirred for 60 min. The 50 mL Teflon container was then placed inside an autoclave reactor which was sealed and placed in an oven at 180 °C for 12 h. The reactor was then allowed to cool to room temperature and the mixture was removed. The white powder was washed with deionized water and anhydrous ethanol three times each, alternating between the two and finishing with anhydrous ethanol. The final product was placed in an oven at 60 °C for 12 h. The obtained white powder was then mortared and kept in a dark desiccator to avoid degradation before use.

### 2.3. Experimental procedure

**Fig. 1:**
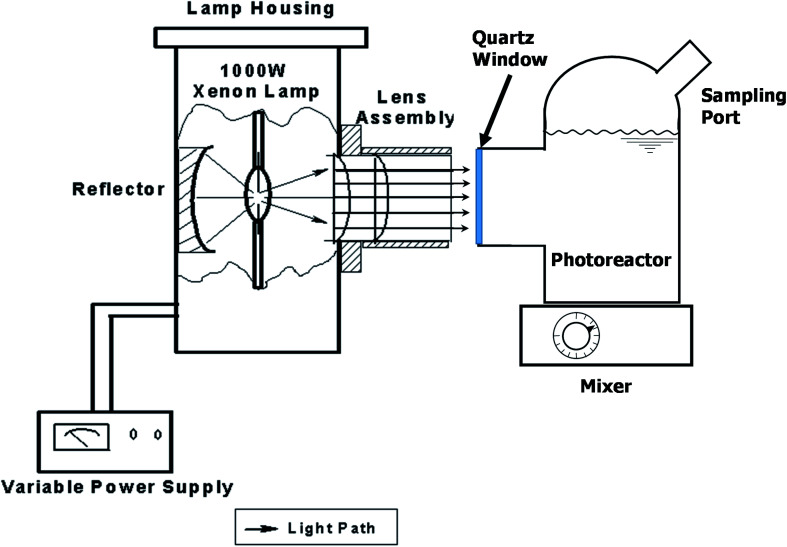
Experimental set up showing the xenon light source system and custom-made batch photoreactor (*Φ*80 mm × 53 mm *H*) equipped with a quartz window (*Φ*36 mm) and an injection/sampling port. Lamp housing and lens assembly are drawn to show the interior components.

The photocatalytic activity of all samples was evaluated under a 1000 W solar simulator xenon arc lamp (model 6269, Oriel Corporation). The photoreactor used was a custom blown 150 mL Pyrex glass vessel with optically clear quartz window, as shown in Fig. 1.

Methyl orange degradation experiments were performed to examine the overall efficiency of the photoreactor in terms of light delivery. Methyl orange stock solution with a concentration of 30 μM was prepared in deionized water and 100 mL of the solution was placed in the batch photoreactor. The solution was mixed in the dark for 15 min after which 0.1 g of photocatalyst was added and mixed for another 15 min prior to illumination. Samples were collected from the photoreactor using a 1 mL plastic syringe and were put through a 0.2 μm PTFE filter to remove any remaining TiO_2_.

For glycerol experiments, 0.0368 g (4 mM) glycerol was added to 100 mL of acetonitrile (or water) in the batch photoreactor. In crude glycerol experiments, a liquid–liquid extraction was performed to separate the glycerol and dissolve it into the solvent. This was done by measuring as received crude glycerol into a sealed polypropylene bottle along with 100 mL of acetonitrile. The mixture was then placed in a sealed shaking oven at 40 °C and 400 rpm for 24 h. The photocatalytic reactions were conducted following the above procedure and samples were collected at prescribed intervals. All experiments were performed at room temperature and in duplicate. Conversion and selectivity of the catalyst was determined using:
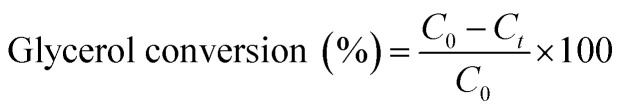

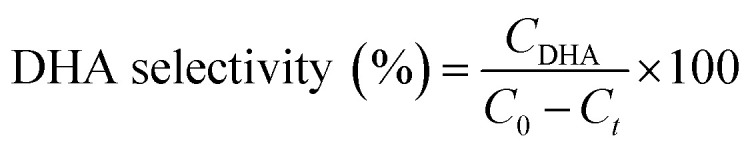
where *C*_0_ is the initial concentration of glycerol; *C*_*t*_ is the concentration of glycerol at time ‘*t*’, and *C*_DHA_ is the concentration of DHA at time ‘*t*’.

Adsorption experiments were conducted at 25 ± 1 °C by adding 100 mL of acetonitrile to a 125 mL polypropylene bottle along with the appropriate amount of glycerol. The mixture was stirred vigorously for 60 min and a ‘before’ sample was collected for comparison. 0.1 g of TiO_2_ was then added and the mixture was stirred for an additional 24 h and an ‘after’ sample was taken. All samples, as well as standards, were passed through a 0.2 μm PTFE filter to remove the remaining photocatalyst particles, if any. The equilibrium adsorption capacity, *q*_e_ (mg g^−1^), of TiO_2_ was calculated by:
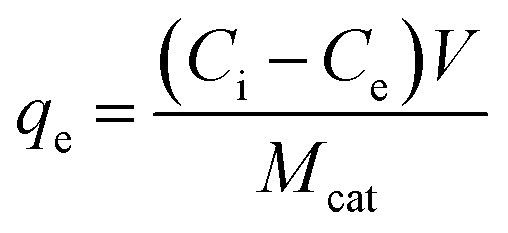
where *C*_i_ (mg L^−1^) and *C*_e_ (mg L^−1^) are the initial and equilibrium concentrations of glycerol, respectively, *V* (L) is the volume of acetonitrile used and *M*_cat_ (g) is the mass of the TiO_2_ photocatalyst that was added to the solution.

Langmuir and Freundlich adsorption models were used to further analyze the adsorption of glycerol on lab-made TiO_2_ and Degussa P25 TiO_2_. For Langmuir isotherm:
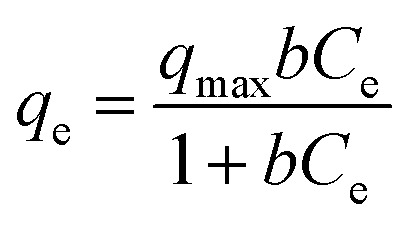
where *q*_max_ (in mg g^−1^) represents the maximum adsorption capacity of TiO_2_ and *b* (in L mg^−1^) is the Langmuir reaction constant which is related to the free energy of adsorption. Similarly, for Freundlich isotherm:*q*_e_ = *K*_f_*C*_e_^1/*n*^where *K*_f_ (mg^1−1/*n*^ L^1/*n*^ g^−1^) is the Freundlich constant that relates to the adsorption capacity of the adsorbent and 1/*n* is the heterogeneity parameter which relates to the adsorption intensity.

### 2.4. Analytical methods

A Hitachi High Technologies SU 8230 FESEM was employed to characterize the morphology of the nanoparticles. The XPS analysis was conducted on a Thermo Fisher Scientific K-Alpha using monochromated Al K-Alpha X-rays with a 400 μm analysis spot. The survey scan was performed at a pass energy of 200 eV and the narrow scan was performed at a pass energy of 50 eV. Phase detection and quantification were conducted using a Rigaku MiniFlex 600 X-ray diffractometer (XRD) using Cu generated X-rays. BET analysis was performed using an Autosorb-1 surface area and pore size analyzer by Quantachrome Instruments. For this, a sample of approximately 0.4 g of the photocatalyst was degassed under vacuum at 300 ± 10 °C for 0.5 h to achieve a pressure of 1.4 Pa before analysis was performed with N_2_ at 77 K.

Samples for scanning transmission electron microscopy (STEM) were prepared by drop casting onto holey carbon grids. High angle annular dark field (HAADF) and bright field (BF) images were captured with a Hitachi HF-3300 scanning transmission electron microscope, operated at 300 kV.

Methyl orange concentration was determined using a UV-Vis spectrophotometer (Hitachi U-3900) based on the absorption peak at 464 nm. Concentration of glycerol and its reaction products were determined using HPLC-MS (Thermo Q-Exactive MS HESI II Dionex Ultimate 3000 UHPLC with a Phenomenex Luna-NH_2_ column (150 mm × 2 mm × 3 μm)). The determination was carried out at 40 °C under constant flow rate of 0.3 μL min^−1^ and injection volume of 10 μL, mobile phase A, 0.1% formic acid in water mobile phase B, 0.1% formic acid in acetonitrile, 20 : 80 A : B for 3 min, 20 : 80 to 80 : 20 over 3 min, 80 : 20 for 3 min, 80 : 20 to 20 : 80 for 1 min and equilibrate for 5 min. To enhance the detection of reaction by-products formed in trace concentrations by HPLC-MS^2^, cryodesiccation was performed to concentrate the samples. 50 mL of the reaction mixture was exposed for 24 h to −85 °C, then transferred to a FreeZone Plus 2.5 L freeze drying system with a vacuum of <0.133 mbar and a temperature of −84 °C for 24 h.

To address matrix issues associated with HPLC-MS analysis of crude glycerol samples, these samples were derivatized with DNPH prior to quantitative analysis. The DNPH was dissolved in 60 °C acetonitrile, stirred for 10 min, cooled to room temperature and filtered to obtain purified DNPH. 2.38 g of DNPH was then dissolved in 10 mL of water and was stirred continuously. 0.1 mL of purified DNPH and 0.138 mL of a prepared 0.036 M HCl solution were added to 0.5 mL of sample containing crude glycerol. The mixture was then placed in a sealed shaking oven set at 40 °C and 200 rpm for 24 h. The solution was then further purified using a HyperSep C18 SPE cartridge purchased from Thermo Scientific. The cartridge was pre-treated with 1 mL methanol then with 1 mL methanol–water solution (7 : 3). The derivatized crude glycerol reaction sample was then added to the cartridge and left for 30 s. The cartridge was then washed twice with 0.5 mL methanol–water solution (9 : 1) and dried for 3 min. The final elution was conducted with 0.5 mL of methanol and collected slowly.^23^ These samples were analyzed using the previously described HPLC-MS using an Aminex HPX-87H column (300 mm × 7.8 mm × 9 μm). The determination was carried out at 60 °C under constant flow rate of 0.6 μL min^−1^ and injection volume of 10 μL. An identical mixture of 3 mM glycerol, 0.2 mM GAD and 0.2 mM DHA in water was analyzed 5 times, the signal reported were all within 10% of each other.

All electrochemical tests were performed using a method similar to those found in literature sources.^24–26^ In a single chamber quartz glass reactor, data acquisition using an electrochemical workstation (CHI660E) with Na_2_SO_4_ (0.1 M) electrolyte solution or acetonitrile solution with a standard three-electrode system. Saturated calomel electrode (SCE) and platinum plate electrode were used as reference electrode and counter electrode, respectively. The catalyst is loaded onto the FTO conductive glass (fluorine doped SnO_2_ transparent conductive glass) and naturally dried to obtain a working electrode. 10 mg of the samples were dispersed in a solution containing 475 μL of deionized water, 475 μL of absolute ethanol and 50 μL of Nafion solution. Then, 10 μL of the above suspension liquid was dropped onto the FTO conductive glass with a fixed exposure area of 0.126 cm^−2^ the loading is almost 0.8 mg cm^−2^. A 300 W Xe lamp with full spectrum was fixed in front of the reactor. The light intensity is approximately 1 mW cm^−2^. Electrochemical impedance spectroscopy (EIS) was conducted at 0.8 V *versus* SCE in a frequency ranging from 1 Hz to 100 kHz with an amplitude of 5 mV. Photocurrent test was conducted at 0 V and 0.8 V *versus* SCE.

## 3. Results and discussion

### 3.1. Characterization of catalysts

**Fig. 2:**
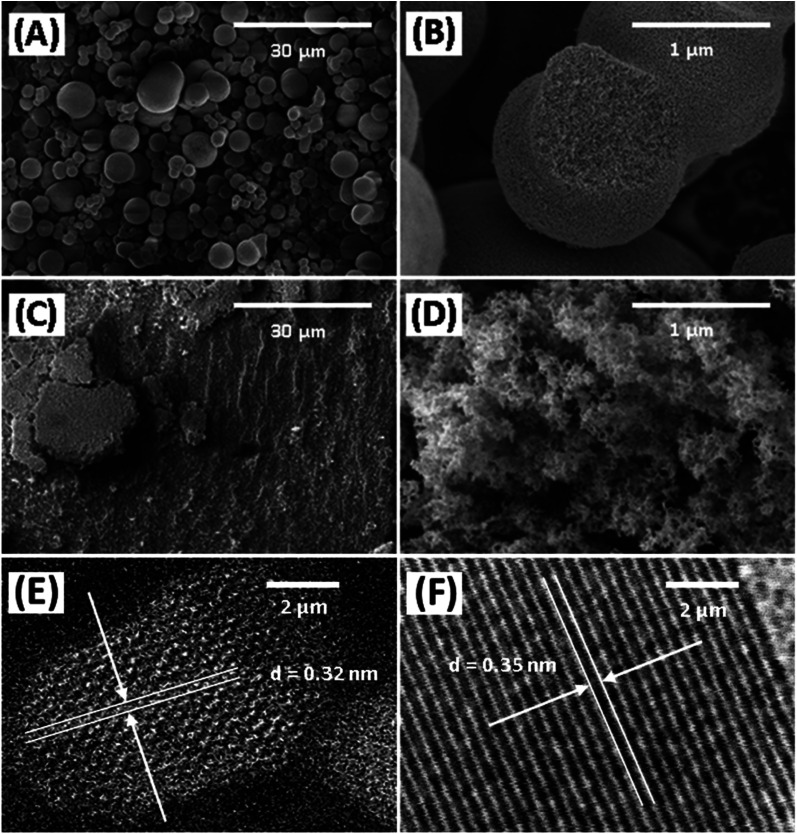
SEM micrographs of lab-made TiO_2_ (A and B) and Degussa P25 TiO_2_ (C and D) highlighting differences in the morphology of agglomerated catalyst particles, and high-angle annular dark field STEM micrographs of lab-made TiO_2_ (E) and Degussa P25 TiO_2_ (F).

**Fig. 3:**
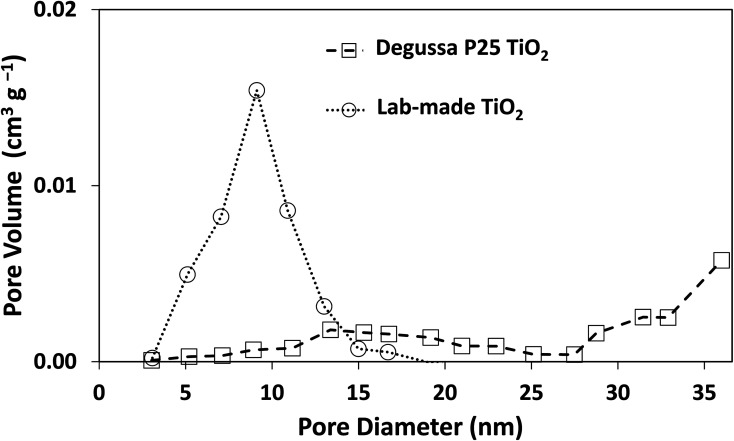
Pore size distribution curves for both Degussa P25 TiO_2_ and lab-made TiO_2_. Nitrogen adsorption–desorption isotherms for both Degussa P25 TiO_2_ and lab-made TiO_2_ are shown in Fig. S.1.†

The morphology of Degussa P25 and lab-made TiO_2_ are shown in Fig. 2 in SEM and STEM images. The STEM image and lattice distance for Degussa P25 TiO_2_ are similar to that found by Wang *et al.*^27^ From STEM images we can also obtain a primary particle size of approximately 10 nm for lab-made TiO_2_ and 30 nm for Degussa P25 TiO_2_. The BET surface area of lab-made TiO_2_ was determined to be 218.5 m^2^ g^−1^ which was significantly higher than that of Degussa P25 TiO_2_, 64.2 m^2^ g^−1^ (shown in Fig. S.1†), as reported in the literature.^28–30^Fig. 3 shows the pore size distribution of both lab-made TiO_2_ and Degussa P25 TiO_2_, the pore size distribution for Degussa P25 TiO_2_ is also similar that found by Wang *et al.*^27^ The pore size distribution of both lab-made TiO_2_ and Degussa P25 TiO_2_ peak at 9 nm and 36 nm, respectively, which is similar to their primary particle size as determined by STEM. This may indicate that the majority of the pores observed in the BET pore size distribution are caused by interparticle pores in the agglomerated TiO_2_ (shown in Fig. 2A–D) rather than pores found inside the individual primary particles of either lab-made TiO_2_ or Degussa P25 TiO_2_.

**Fig. 4:**
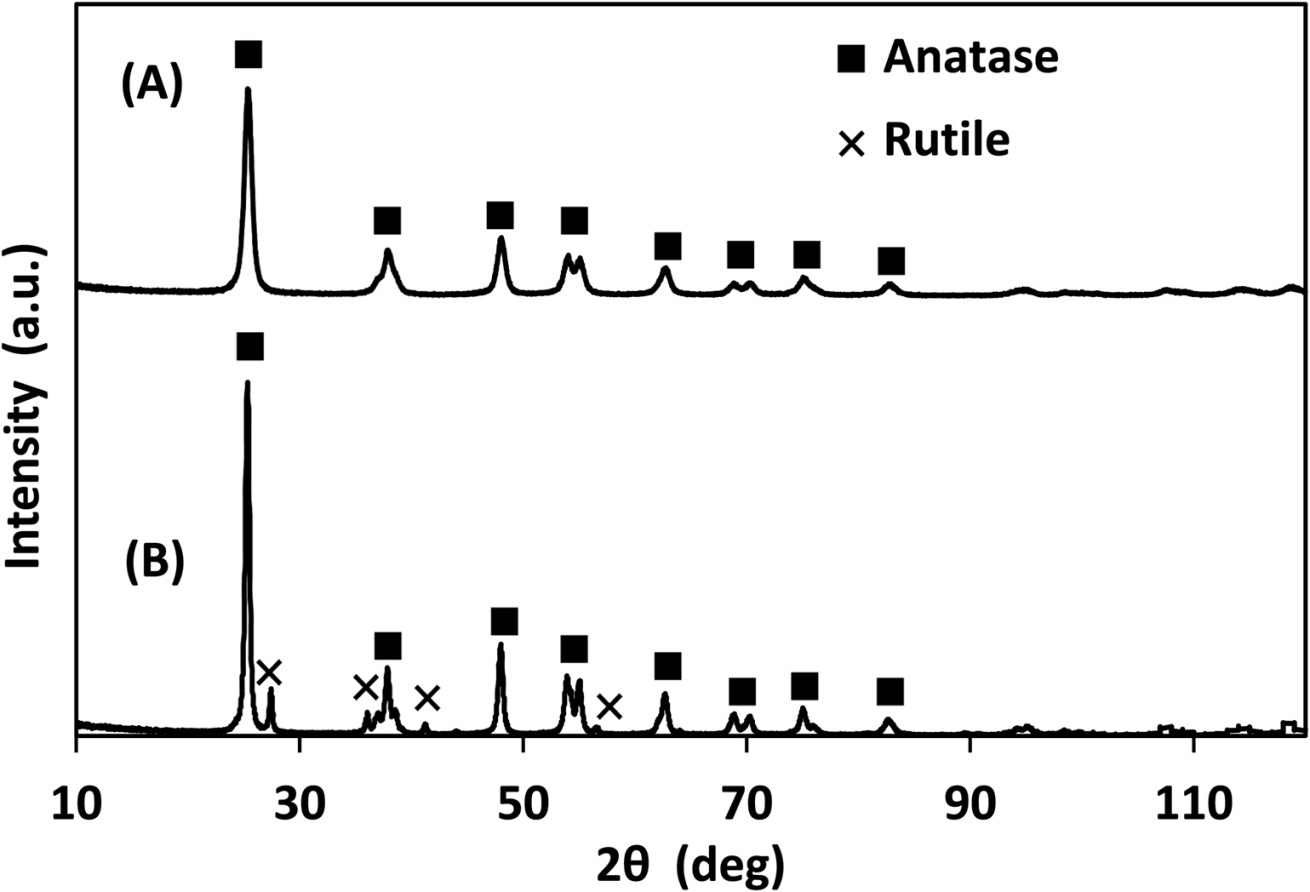
XRD of (A) lab-made TiO_2_ as observed, best fit to 100% anatase, and (B) Degussa P25 TiO_2_ best fit to 88.3% anatase and 11.6% rutile.

The X-ray photoelectron spectra (XPS) of the photocatalysts are provided in Fig. S.2–S.4† and their elemental analysis is summarized in Table 1. Lab-made TiO_2_ had significantly lower carbon content than Degussa P25 TiO_2_, likely due to the high affinity of P25 for the adsorption of volatile organic compounds.^31^ According to the XRD data presented in Fig. 4, the lab-made TiO_2_ was found to be 100% anatase while Degussa P25 TiO_2_ was 88.3% anatase and 11.6% rutile. It has been reported that the anatase form of TiO_2_ has a higher photocatalytic activity than its rutile form.^32–34^

**Table 1:** Elemental surface analysis of lab made and Degussa P25 TiO_2_ by XPS

	Ti	O	C	N	Cl	W	Si
Lab-made	27.30	55.24	16.39	0.65	0.29	0.13	—
P25	16.15	37.30	45.95	0.13	0.16	—	0.31

**Fig. 5:**
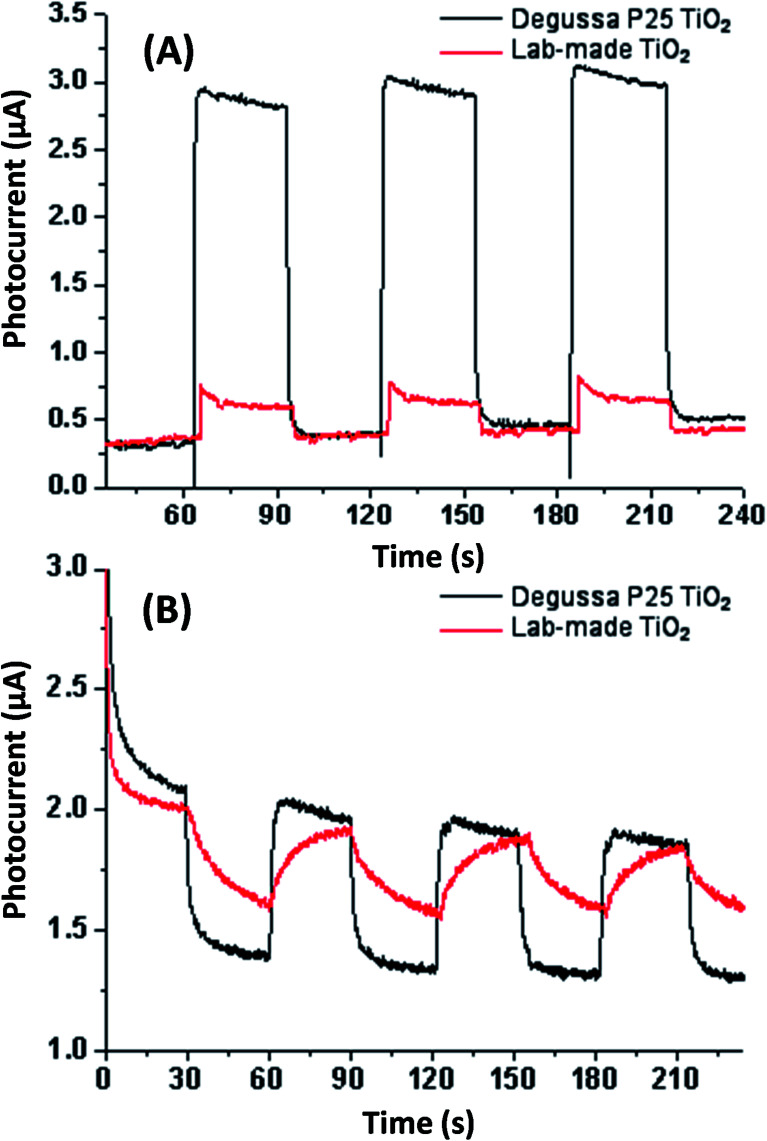
Photocurrent testing in aqueous sodium sulfate solution (A) and acetonitrile (B) both without applied bias potential. See Fig. S.5† for photocurrent and EIS testing in aqueous sodium sulfate solution with applied bias potential.

Electrochemical testing was conducted in aqueous sodium sulfite solution and acetonitrile for both Degussa P25 and lab-made TiO_2_ as shown in Fig. 5. This shows that in water Degussa P25 TiO_2_ has a higher photocurrent and lower resistance, this is consistent with Chen *et al.* who also report that their pure anatase TiO_2_ shows a lower photocurrent than Degussa P25 TiO_2_.^35^ In acetonitrile the difference between Degussa P25 TiO_2_ and lab-made TiO_2_ photocurrent and resistance was significantly less. This can be partially attributed to the change of solvent being significantly less conductive.

Similar to other research using dye degradation to measure photocatalytic activity, we performed control experiments using 30 μM of methyl orange with 1 g L^−1^ of Degussa P25 TiO_2_ which reached total degradation at 120 min (Fig. S.6†).^36^ Previous literature using a UV lamp (300 W, 375 nm) achieved the complete degradation of methyl orange after 300 min with identical amounts of methyl orange and Degussa P25 TiO_2_.^37^ It was observed that the degradation of methyl orange with lab-made TiO_2_ was within 10% of that observed with Degussa P25 (Fig. S.6†), suggesting that the two photocatalysts had similar activities under these experimental conditions. Given the larger BET surface area and the anatase content of lab-made TiO_2_, this result suggests that methyl orange degradation in these experiments was likely limited by the light intensity. Considering the above findings, lab-made TiO_2_ was selected for glycerol conversion in the subsequent sections.

### 3.2. Photocatalytic conversion of glycerol in acetonitrile

**Fig. 6:**
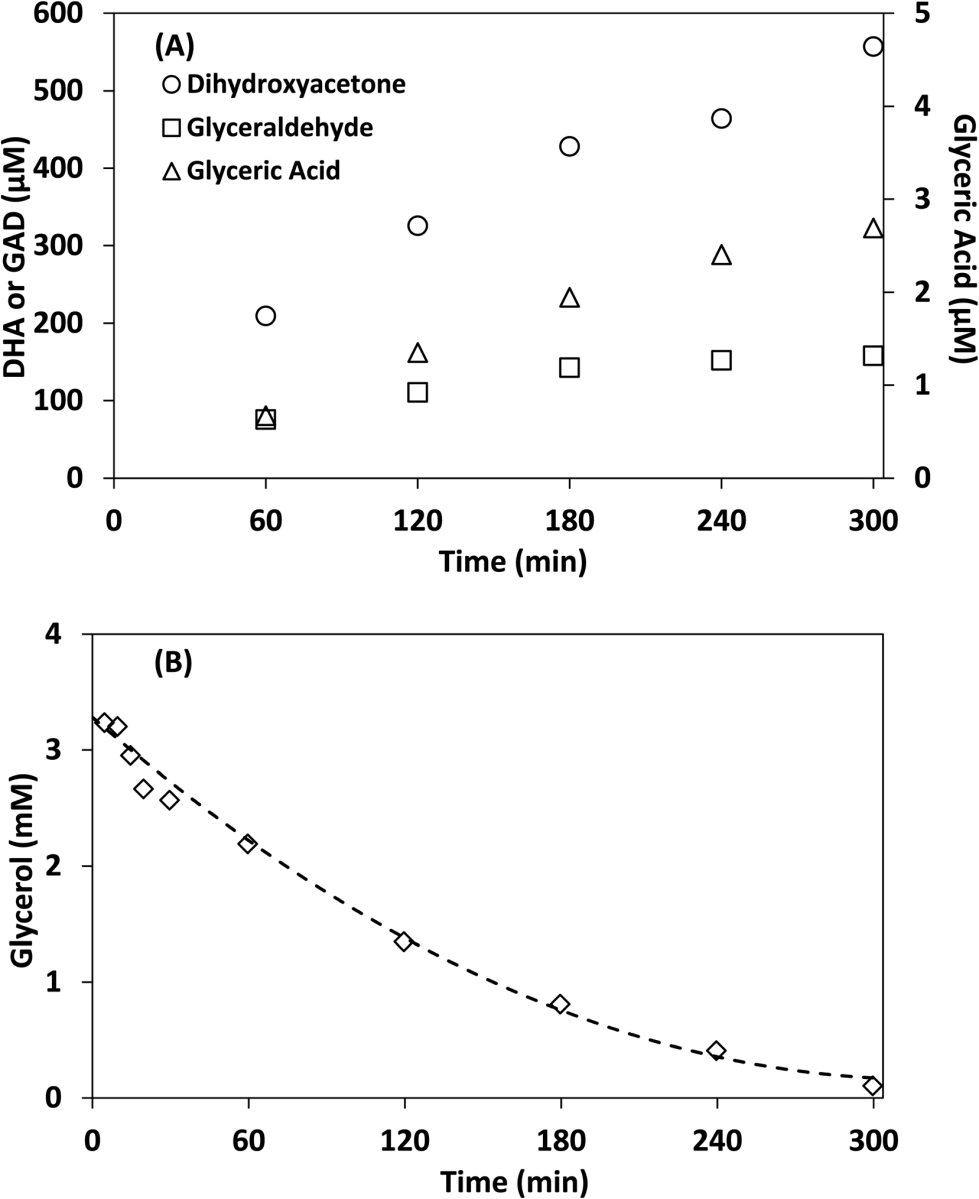
Photocatalytic reaction of glycerol in acetonitrile under simulated solar light. (A) The concentration of DHA, GAD, glyceric acid and (B) concentration of glycerol over time. Acetonitrile: 100 mL, glycerol concentration: 4 mM, lab-made TiO_2_: 1 g L^−1^.

Control experiments showed no measurable decrease in glycerol concentration in the absence of irradiation or TiO_2_ in acetonitrile after 300 min, additionally no species were detected by HPLC-MS in the absence of glycerol after 300 min. Fig. 6 shows the concentration of DHA, GAD, glyceric acid and glycerol over time using lab-made TiO_2_. The half-life in this experiment was found to be 75 min, with 96.8% of glycerol consumed after 300 min. The selectivity of DHA remained relatively unchanged after 60 min and was calculated to be 17.8% at 300 min. The DHA : GAD ratio increased continuously over time and reached a maximum of 3.5 as the GAD concentration appeared to level off after about 180 min. The presence of glyceric acid suggests that GAD was likely oxidized further during the process. In contrast, when using water as the reaction medium after 300 min, glycerol conversion was only 36.1% with a half-life of 467 min (Fig. S.7†) and DHA selectivity was 14.7%. Similar results were obtained for Degussa P25 TiO_2_ where glycerol conversion increased by changing the reaction medium from water to acetonitrile (Fig. S.7 and S.8†). For comparison, Augugliaro *et al.* reported the lowest half-life for Degussa P25 TiO_2_ to be 10.5 h for 10 mM glycerol with 0.2 g L^−1^ of Degussa P25 TiO_2_ in water.^13^ They found that the DHA : GAD ratio and DHA selectivity varied with glycerol and TiO_2_ concentrations, with the highest DHA selectivity of 8% recorded at 100 mM of glycerol and 0.4 g L^−1^ of Degussa P25 TiO_2_.

**Fig. 7:**
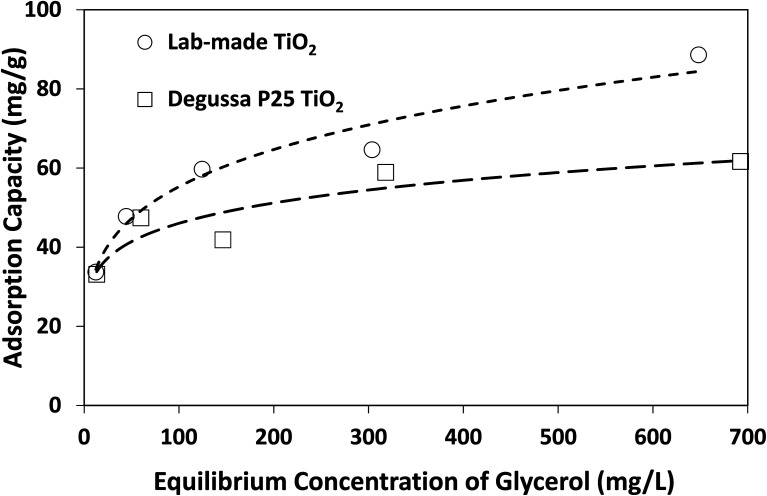
Adsorption isotherms of glycerol on lab-made TiO_2_ and Degussa P25 TiO_2_ in acetonitrile at room temperature.

The above findings highlight the significance of reaction medium in promoting the selective photocatalytic production of DHA from glycerol. The change of solvent from water to acetonitrile is a transition from a more polar to a less polar medium (at 25 °C, the relative permittivity of water and acetonitrile are 78.4 and 35.9, respectively^38^). Adsorption isotherms as well as Langmuir and Freundlich parameters provided in Fig. 7 and Table 2 show that lab-made TiO_2_ had a higher adsorption capacity for glycerol in acetonitrile than Degussa P25 TiO_2_. However, in water and under similar conditions, no measurable adsorption of glycerol on photocatalysts was detected. In fact, control experiments conducted using a 4 mM solution of glycerol in water with 10 g L^−1^ TiO_2_ (*i.e.* 10 times higher than catalyst concentration in the photoreactor) still showed no detectable adsorption on either lab-made or Degussa P25 TiO_2_. In acetonitrile and with only 1 g L^−1^ catalyst, glycerol adsorption on Degussa P25 and lab-made TiO_2_ were 15.6% and 17.5%, respectively. Hence, in an aqueous slurry of TiO_2_, water molecules are more likely to adsorb on the photocatalyst to form oxidative species that subsequently react with glycerol. In the presence of acetonitrile, however, glycerol can adsorb and react directly on the surface of TiO_2_ increasing the glycerol conversion. This can explain the finding that both the lab-made and Degussa P25 TiO_2_ exhibited enhanced conversion of glycerol in acetonitrile (97.8% and 66.8% at 300 min, respectively) compared to water (36.1% and 50.3% at 300 min, respectively).

**Table 2:** Langmuir and Freundlich model parameters for adsorption of glycerol on TiO_2_ in acetonitrile

Photocatalyst	Langmuir			Freundlich		
	*q*_max_ (mg g^−1^)	*b* (L mg^−1^)	*R* ^2^	*K*_f_ (mg^1−1/*n*^ L^1/*n*^ g^−1^)	1/*n*	*R* ^2^
Lab-made TiO_2_	70.42	0.0712	0.911	19.47	0.2266	0.973
Degussa P25 TiO_2_	54.65	339.41	0.776	22.77	0.1529	0.844

### 3.3. Analysis of reaction by-products

**Fig. 8:**
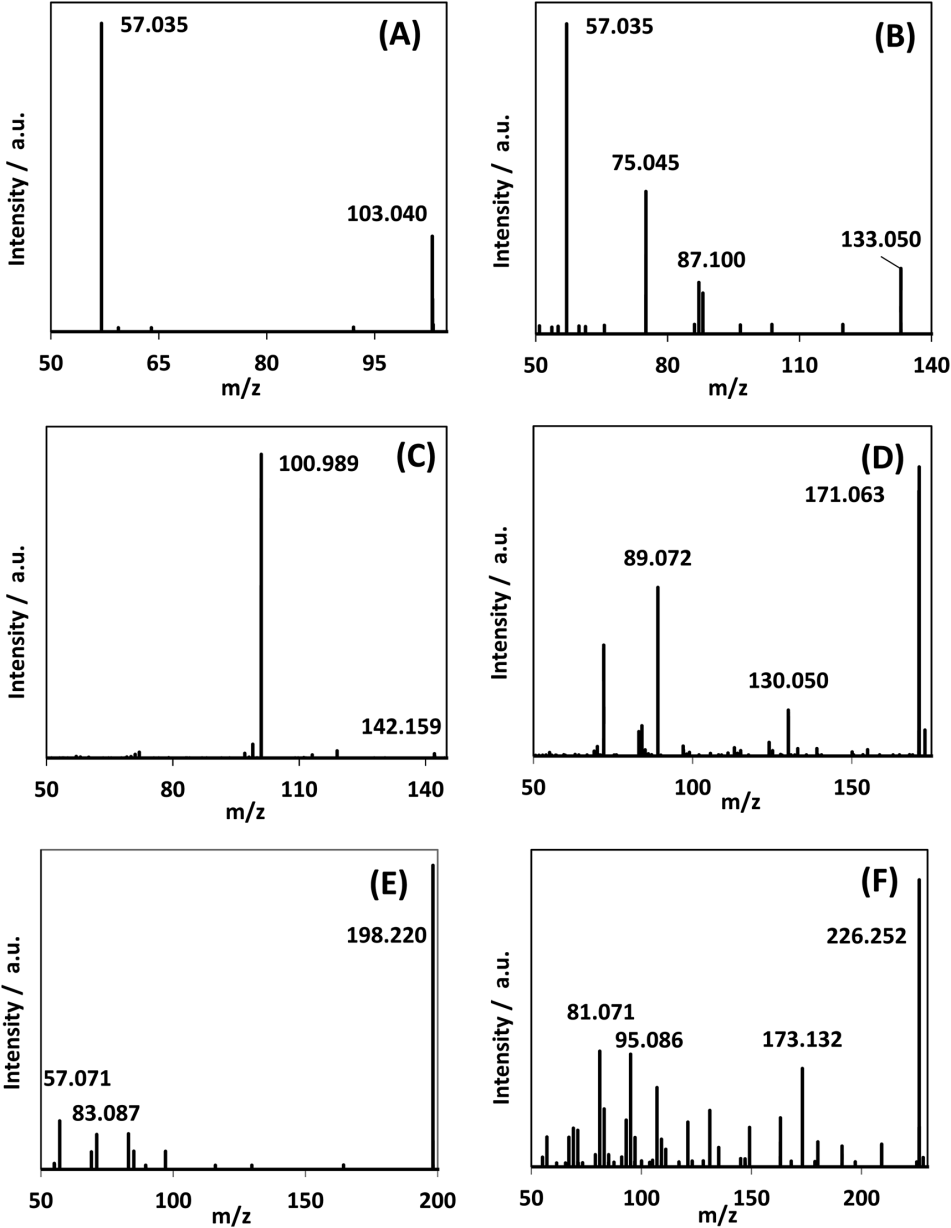
MS/MS spectra of reaction mixture for species observed in the reaction medium after 300 min reaction time. (A)–(C) were assigned as C_4_H_6_O_3_, C_5_H_8_O_4_, and C_9_H_19_N. Fig. S.9† shows the generation of these species over time.

In addition to DHA, GAD and glyceric acid, HPLC-MS spectra showed the generation of other species with mass-to-charge ratios of 103.040, 133.050, 142.159, 171.063, 198.220, and 226.252 (Fig. 8). By assigning elemental compositions restricted to C, N, O and H and with the assumption that all species were protonated during ionization (based on the acidic solvent used during analysis and the use of positive ion mode in the mass spectrometry performed), 103.040, 133.050, and 142.159 *m*/*z* ratios were assigned to C_4_H_6_O_3_, C_5_H_8_O_4_ and C_9_H_19_N, respectively. Formation of such species, with a molecular mass larger than glycerol, is indicative of addition reactions.

**Fig. 9:**
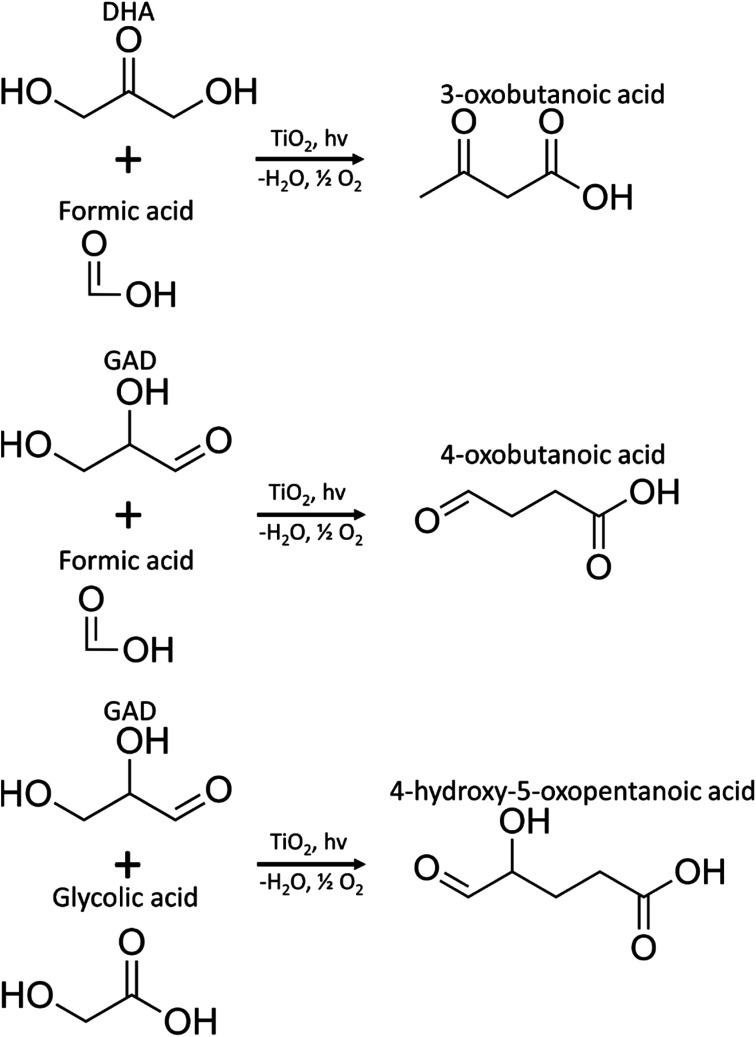
Proposed pathways for the production of C_4_H_6_O_3_ (3-oxobutanoic acid or 4-oxobutanoic acid) and C_5_H_8_O_4_ (4-hydroxy-5-oxopentanoic acid) species.

MS/MS of the singly protonated C_4_H_6_O_3_ parent ion in Fig. 8A showed a strong *m*/*z* 57.035 fragment ion that can be labelled as C_3_H_5_O. Formation of this ion is due to a neutral loss of CH_2_O_2_ that could be either due to the loss of formic acid or loss of CO_2_ and H_2_, both suggesting the presence of a carboxylic acid functional group in this compound. Accordingly, this compound was identified as likely to be either 3-oxobutanoic acid or 4-oxobutanoic acid. Similarly, the singly protonated C_5_H_8_O_4_ parent ion in Fig. 8B showed a *m*/*z* 57.035 fragment ion, indicating a neutral loss of C_2_H_4_O_3_. The *m*/*z* 75.045 fragment ion is labelled as C_3_H_7_O_2_, suggesting a neutral loss of C_2_H_2_O_2_. The neutral loss from *m*/*z* 75.045 to *m*/*z* 57.035 matches that of water, which indicates the presence of a hydroxyl or a carboxylic acid functional group. The *m*/*z* 87.100 fragment ion is likely C_4_H_7_O_2_ indicating a neutral loss of CH_2_O_2_ similar to the C_4_H_6_O_3_ species this could be formic acid or a loss of CO_2_ and H_2_, which suggests the presence of a carboxylic acid functional group. Furthermore, given the presence of the *m*/*z* 57.035 fragment ion in the MS/MS of both the C_4_H_6_O_3_ and C_5_H_8_O_4_ species, it is possible that they share the same structure for the C_3_H_4_O portion. Accordingly, this compound was identified as likely to be 4-hydroxy-5-oxopentanoic acid. The proposed structures and possible reaction pathways for both C_4_H_6_O_3_ and C_5_H_8_O_4_ are shown in Fig. 9. However, no definitive structure can be proposed for the other species.

### 3.4. Reaction mechanism

The adsorption mechanism of glycerol could potentially explain the higher production of DHA over GAD in this process. Glycerol contains two primary hydroxyl groups and one secondary hydroxyl group. From a statistical and steric perspective, the likelihood of primary hydroxyl adsorption is favoured over secondary hydroxyl adsorption. Similar to Hirasawa's mechanism,^10^ given that the vicinal hydroxyl group to the adsorbed hydroxyl group undergoes oxidation, the production of DHA is favoured over GAD. Conversely, without adsorption, the two primary hydroxyl groups are statistically favoured to undergo oxidation relative to the single secondary hydroxyl group, thereby favouring the production of GAD over DHA.

**Fig. 10:**
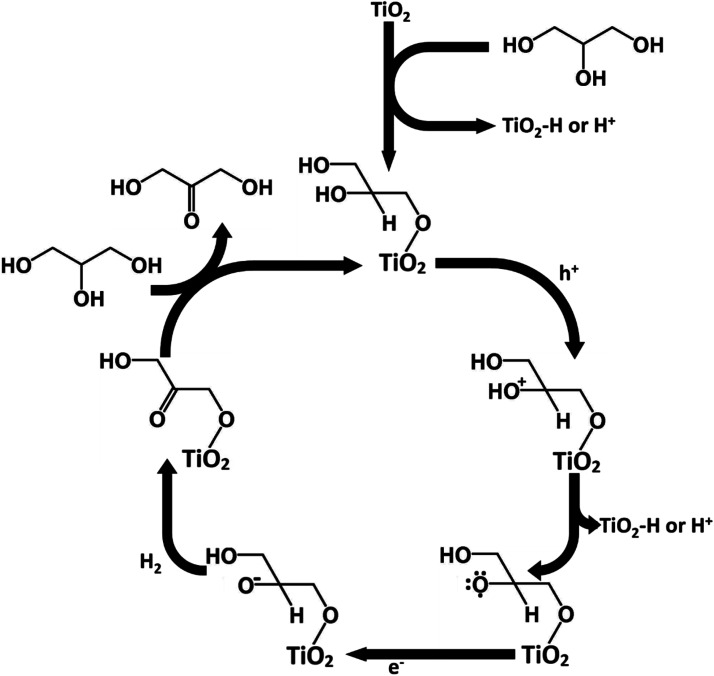
Proposed mechanism for DHA production.

Following Hirasawa, a possible mechanism for the oxidation of glycerol to DHA in acetonitrile has been proposed in Fig. 10. The process begins by the chemisorption of glycerol's primary hydroxyl group. This is accompanied by the loss of a hydrogen which either adsorbs onto the TiO_2_ surface or is released into the solvent. This would be followed by the oxidation of the secondary hydroxyl group on glycerol by losing an electron to fill a hole in the TiO_2_ valence band and the loss of a hydrogen ion or a hydrogen adsorbed to TiO_2_. The glycerol radical would then be reduced to DHA by an electron being donated to the glycerol and another hydrogen being lost to potentially form hydrogen gas. The process would then be continued by glycerol chemisorbing and DHA desorbing.

From the mechanism proposed in Fig. 10, the reason for a higher selectivity towards DHA over GAD would be due to two factors: (a) the steric favourability of adsorbing a primary hydroxyl group over the secondary hydroxyl group, and (b) the presence of two primary hydroxyl groups compared with only one secondary hydroxyl group. This would also imply that selectivity to DHA might be improved if the surface were modified to increase steric inhibition so that GAD production was discouraged. Additionally, as discussed earlier, adsorption is more strongly encouraged in acetonitrile than in water due to its lower relative permittivity. Previous studies involving photocatalytic oxidation of alkanes and primary and secondary alcohols in aqueous medium suggest that the production of hydrogen involves homo- or heterolysis of C–H or O–H bonds with the substrate directly adsorbed to the catalyst,^39^ suggesting that in acetonitrile the oxidation of glycerol may also produce hydrogen.^40,41^

### 3.5. Photocatalytic conversion of crude glycerol

To illustrate the practical utility of the above process, photocatalytic conversion of crude glycerol derived from biodiesel production was examined following a liquid–liquid extraction step. The extraction step was necessary due to the inability to mix crude glycerol and acetonitrile under the same level of agitation as the analytical grade (pure) glycerol used in the previous sections. LC-MS analysis showed that an extraction efficiency of 65% by weight was achieved in these experiments. As discussed in the experimental section, the analysis of the crude glycerol reaction mixture necessitated derivatization using 2,4-dinitrophenylhydrazine (DNPH) prior to LC-MS analysis, because underivatized samples suffered from poor separation from impurities in the crude glycerol which interfered with quantification.

**Fig. 11:**
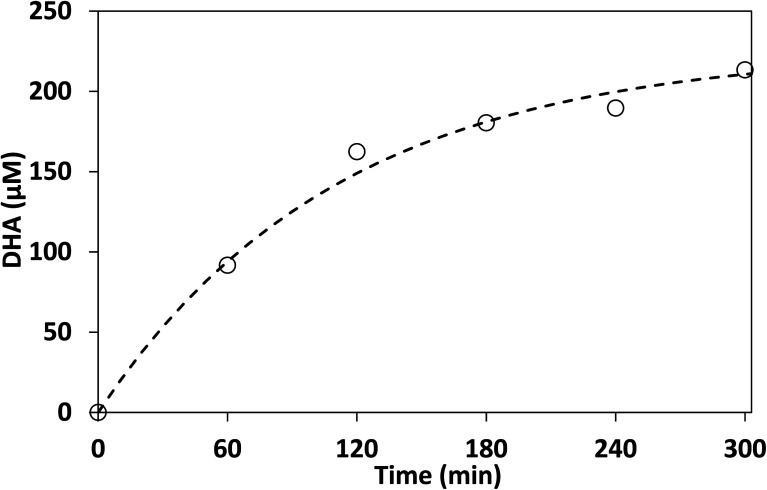
Photocatalytic production of DHA from biodiesel-derived crude glycerol in acetonitrile. Dashed line represents the best fit to first order kinetics model. Acetonitrile: 100 mL, crude glycerol: 0.53 g L^−1^, lab-made TiO_2_: 1 g L^−1^.

The results shown in Fig. 11 indicate that the reaction proceeded in a similar manner to that of the pure glycerol. However, the selectivity to DHA using crude glycerol was found to be 5.7% (assuming all glycerol was consumed, glycerol is not derivatized by DNPH) this is lower than for pure glycerol, possibly due to the other components in crude glycerol poisoning the photocatalyst. An additional sample was taken at 360 min and the concentration of DHA was found to be only 1% higher than at 300 min indicating that DHA conversion reached its maximum after about 300 min.

## 4. Conclusions

Photocatalytic conversion of glycerol to DHA in acetonitrile resulted in higher glycerol conversion and DHA selectivity compared to the reaction in aqueous solution. With an initial glycerol concentration of 4 mM and 1 g L^−1^ of lab-made TiO_2_ and after 300 min irradiation, glycerol conversion and DHA selectivity in acetonitrile were 96.8% and 17.8%, respectively, that were significantly higher than those observed in water, namely 36.1% and 14.7%. The half-life of glycerol was 6.2 times shorter in acetonitrile compared to reaction in water. The higher glycerol conversion and selectivity in acetonitrile may be explained by glycerol adsorption to TiO_2_ in acetonitrile that does not appear to occur in water.

The proposed method of generating DHA from glycerol was successfully applied to crude glycerol generated from biodiesel production with an overall yield of 5.7%. This research offers a significant pathway to generate high value DHA from glycerol while enhancing the environmental friendliness of the biodiesel production process.

## Conflicts of interest

There are no conflicts of interest to declare.

## Supplementary Material

RA-010-C9RA09434B-s001
