# Supplementary material for: Photocatalytic production of dihydroxyacetone from glycerol on TiO2 in acetonitrile
Source: RSC Adv. 2020 Jan 30;10(9):4956–68. doi: 10.1039/c9ra09434b (PMC9049670; doi:10.1039/c9ra09434b)
Supplement: RA-010-C9RA09434B-s001 [file RA-010-C9RA09434B-s001.pdf]

Electronic Supplementary Material (ESI) for RSC Advances.
This journal is © The Royal Society of Chemistry 2020

Photocatalytic Production of Dihydroxyacetone from Glycerol on TiO_2 in Acetonitrile

Alexander Luis Imbault, Jianyu Gong and Ramin Farnood

Supplementary Data

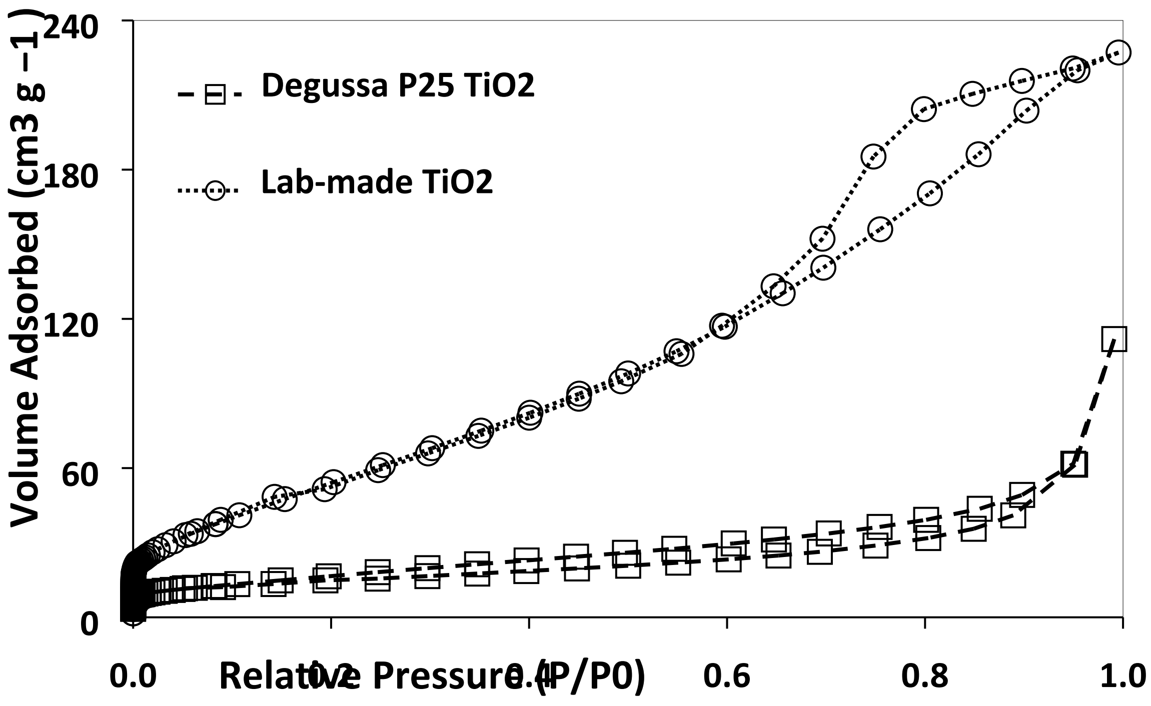

Fig. S. 1. Nitrogen adsorption–desorption isotherms for both Degussa P25 TiO_2 and lab-made TiO_2.

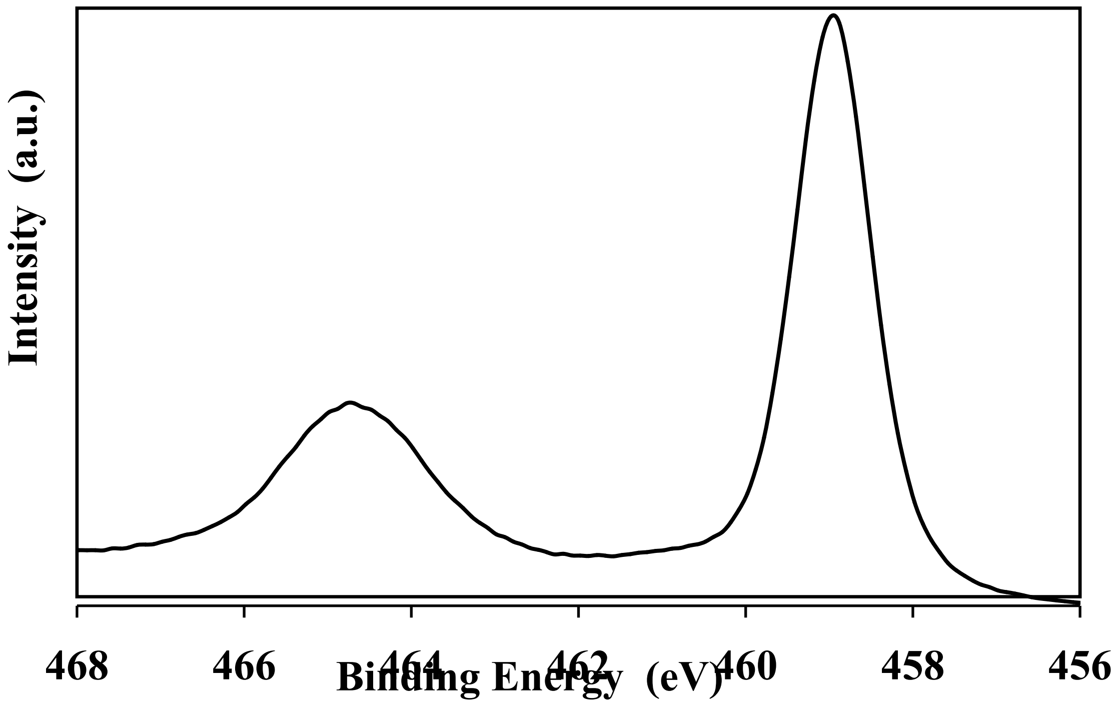

Fig. S. 2. High resolution XPS spectrum of lab-made TiO_2 Ti 2p3/2 at 458.88 eV and Ti 2p1/2 464.68 eV to confirm synthesis of lab-made TiO_2.

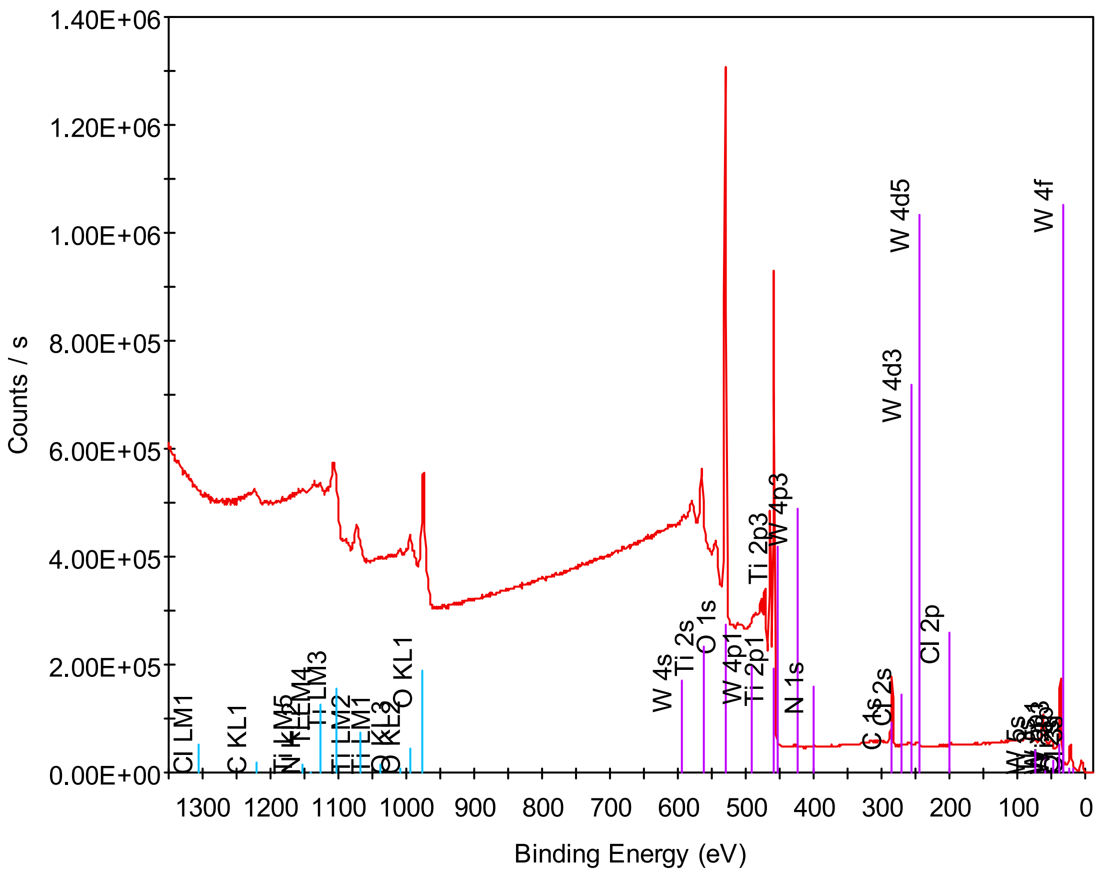

Fig. S. 3. Broad scan XPS of lab-made TiO_2 quantified to contain 27.30% Ti, 55.24% O, 16.39% C, 0.65% N, 0.29% Cl and 0.13% W (atomic percent).

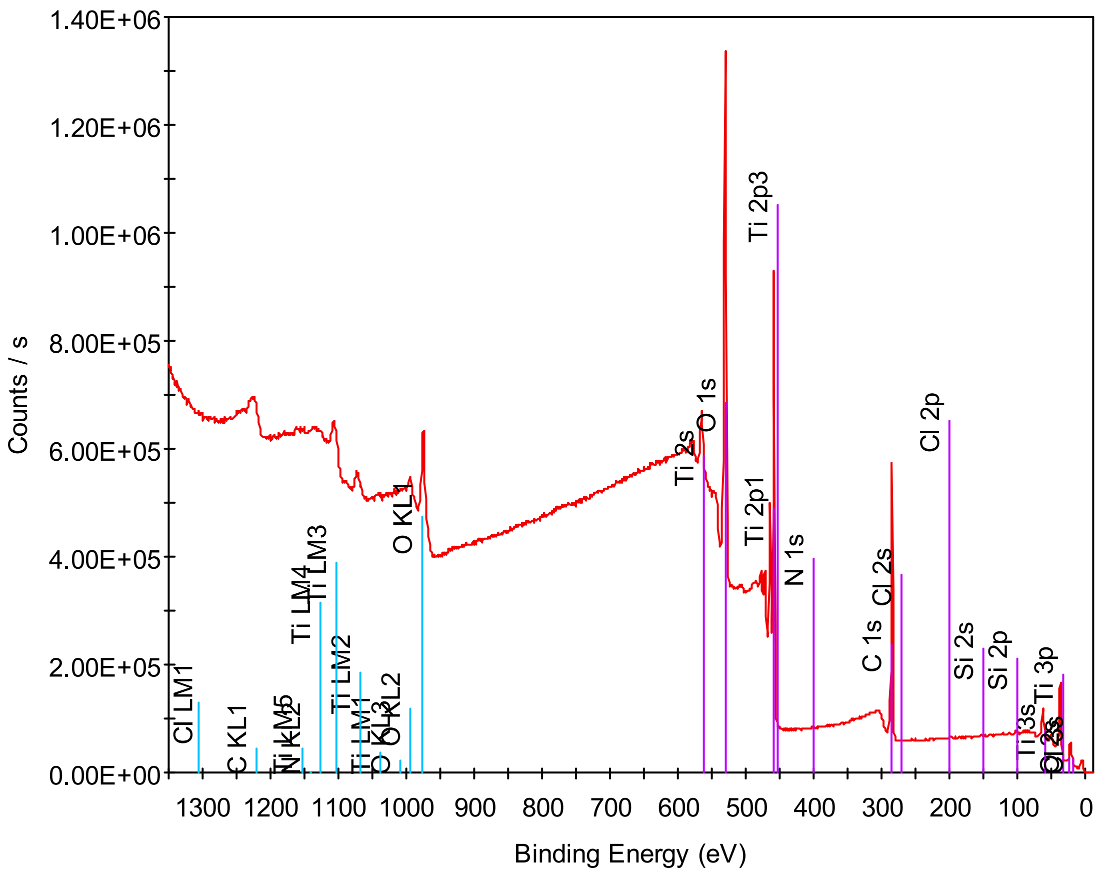

Fig. S. 4. Broad scan XPS of as received Degussa P25 TiO_2 quantified to contain 16.15% Ti, 37.30% O, 45.95% C, 0.13% N, 0.16% Cl and 0.31% Si (atomic percent).

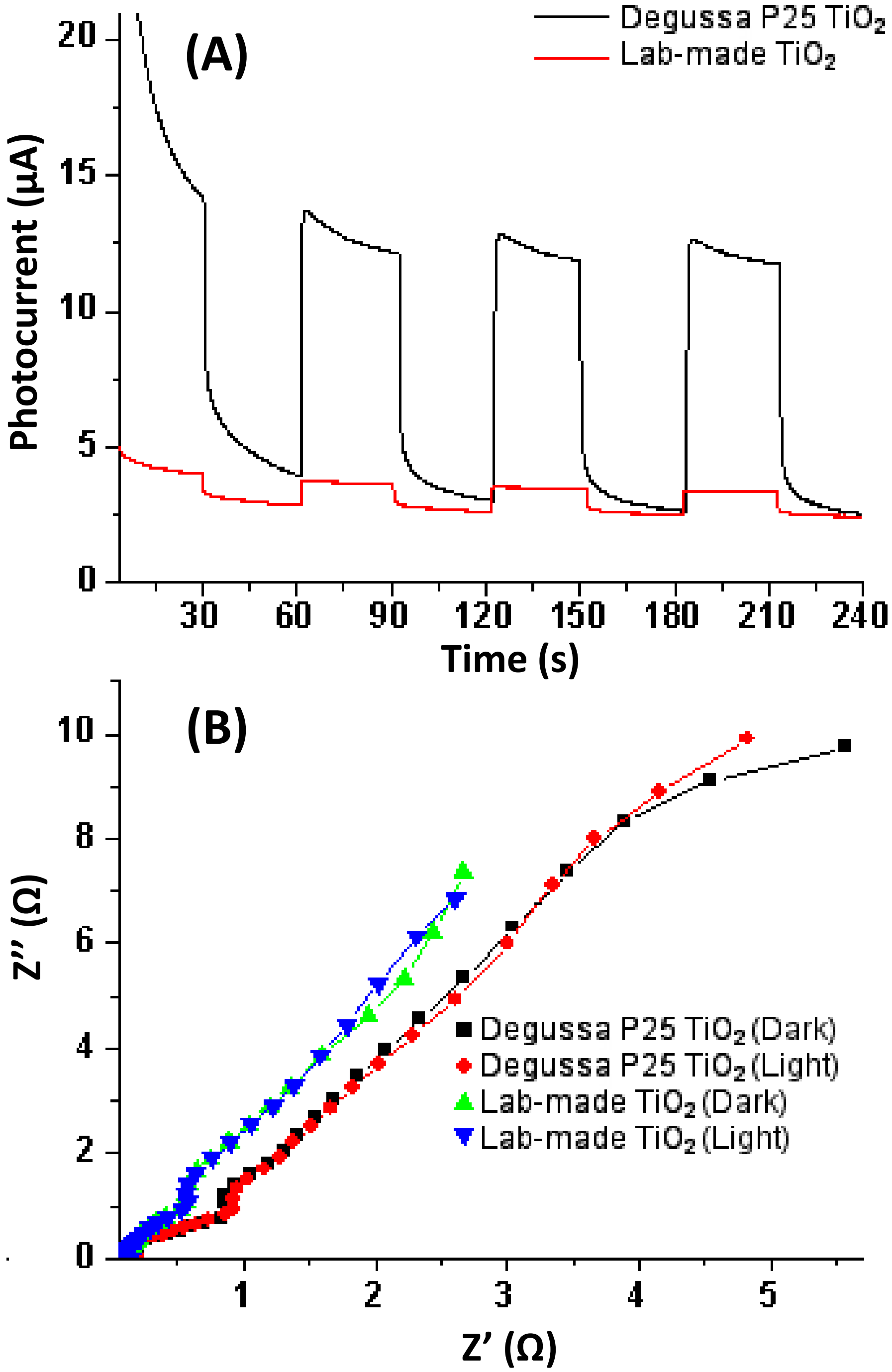

Fig. S. 5. Photocurrent testing in aqueous sodium sulfate solution with applied bias potential (0.8 V, Ref) (A), and EIS test in sodium sulfate solution with applied bias potential (0.8V, Ref) (B).

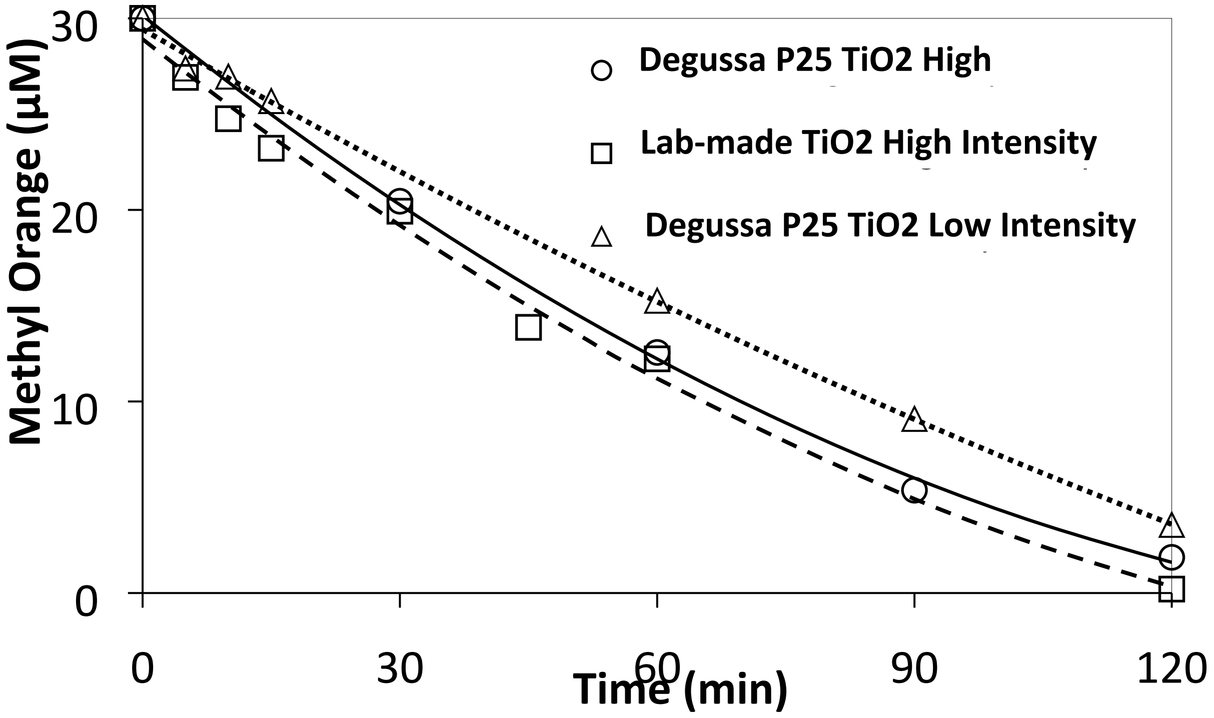

Fig. S. 6. Comparison of Degussa P25 TiO_2 and lab-made TiO_2 for the photocatalytic degradation of methyl orange in water under high and low intensity simulated solar light. Water: 100 mL, initial methyl orange concentration: 30 µM, Degussa P25 TiO_2 or lab-made TiO_2: 1 g/L.

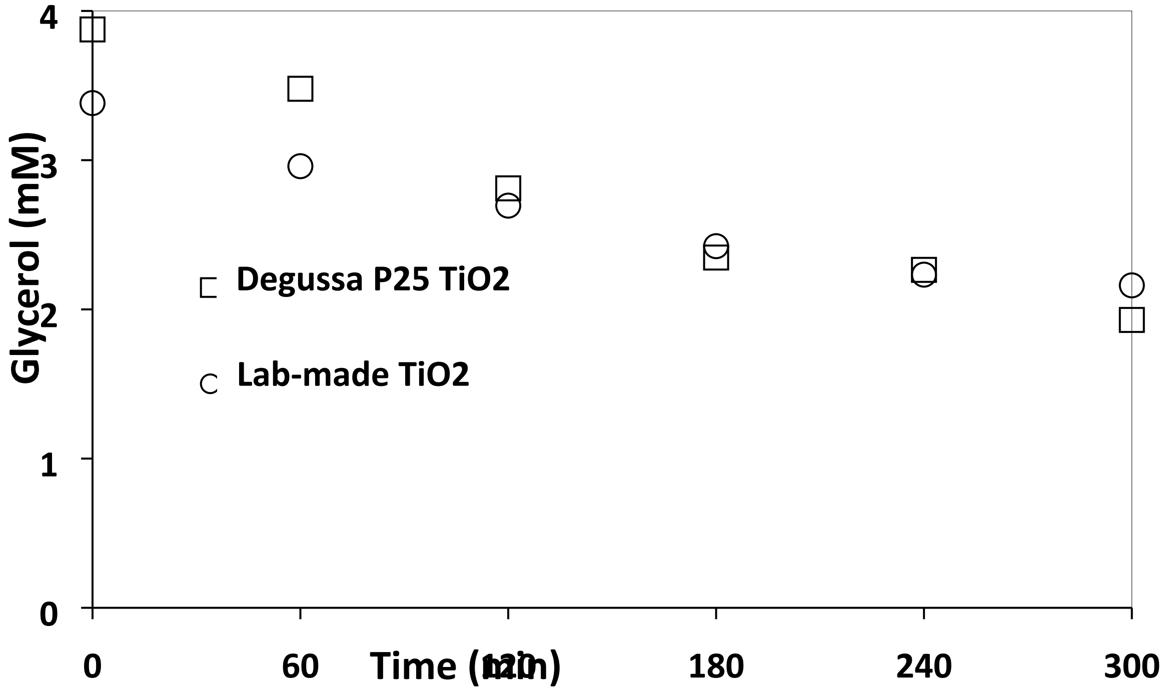

Fig. S. 7. Degradation of glycerol in water with Degussa P25 TiO_2 or lab-made TiO_2 under low intensity simulated solar light. Water: 100 mL, glycerol concentration: 4 mM, Degussa P25 TiO_2 or lab-made TiO_2: 1 g/L.

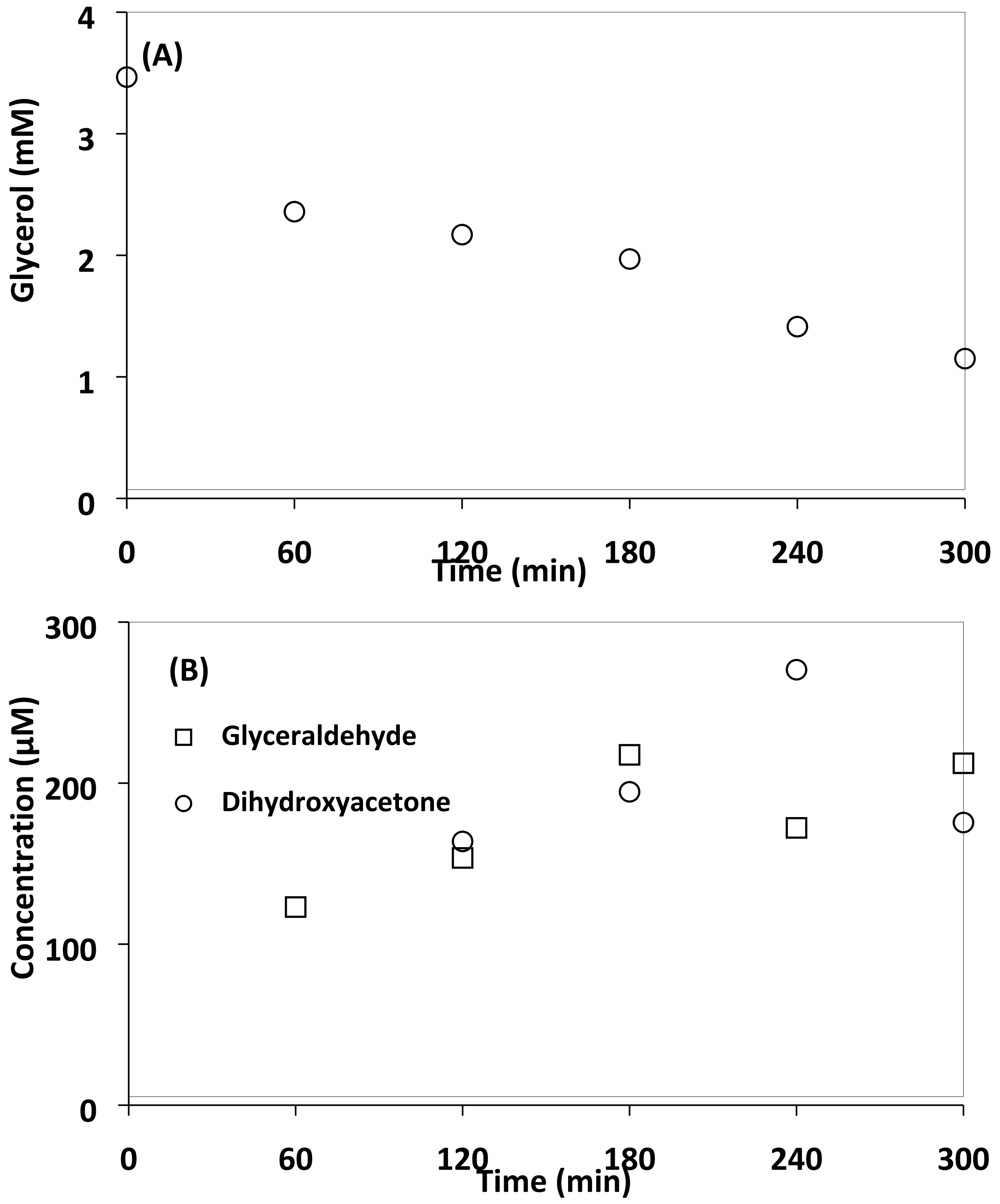

Fig. S. 8. (A) Degradation of glycerol, and (B) generation of dihydroxyacetone (DHA) glyceraldehyde (GAD) in acetonitrile with Degussa P25 TiO_2 under low intensity simulated solar light. Acetonitrile: 100 mL, glycerol concentration: 4 mM, Degussa P25 TiO_2: 1 g/L.

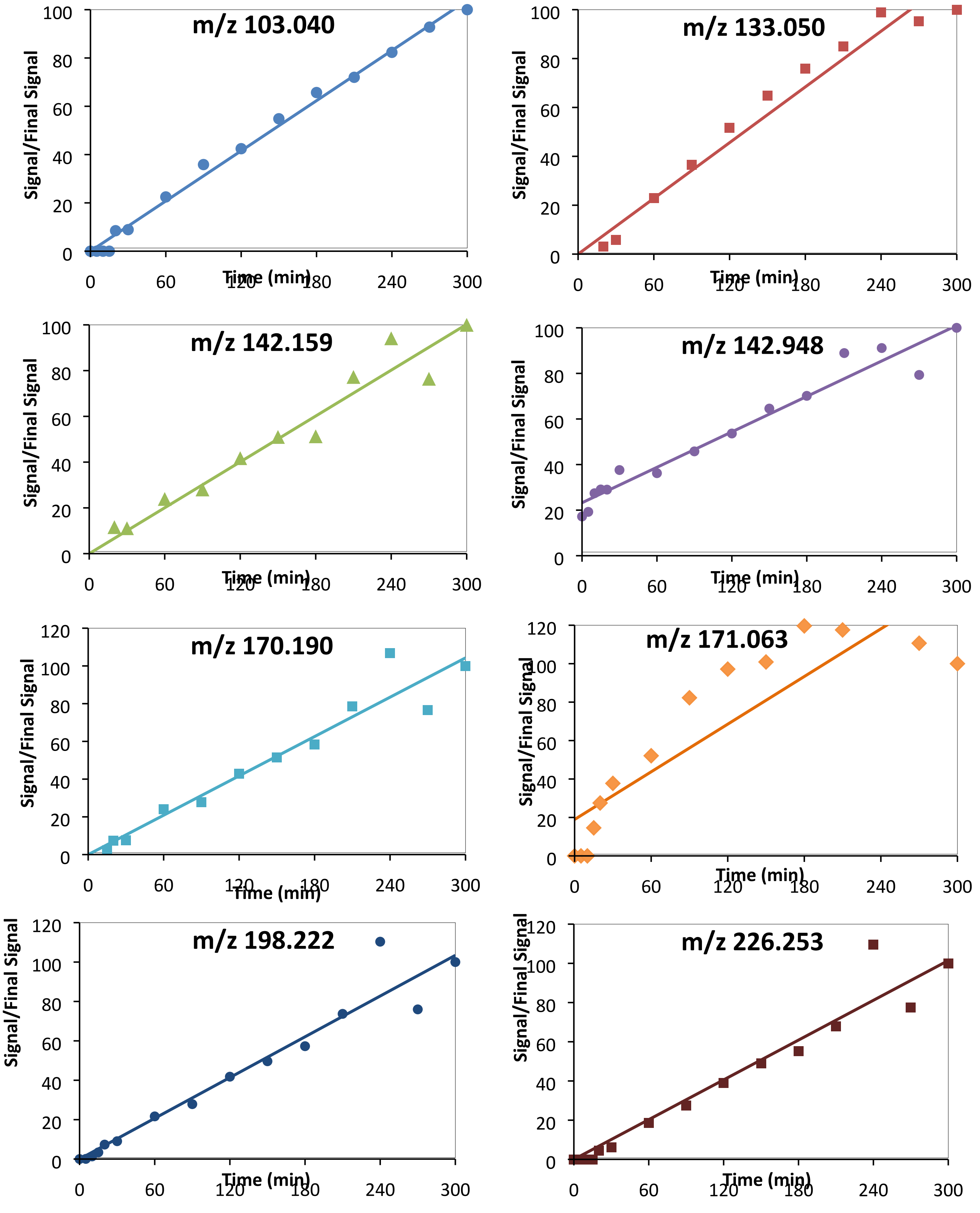

Fig. S. 9. The HPLC-MS signal showing the generation of various species over time during the photocatalytic reaction of glycerol in acetonitrile under solar simulated light.
